# Perceptions of a Transitional Care Model for Older Adults With Multimorbidity and Depressive Symptoms

**DOI:** 10.1093/geroni/igaa057.828

**Published:** 2020-12-16

**Authors:** Maureen Markle-Reid, Carrie McAiney, Rebecca Ganann, Carly Whitmore

**Affiliations:** 1 McMaster University, Hamilton, Ontario, Canada; 2 University of Waterloo, Waterloo, Ontario, Canada

## Abstract

Transitioning from hospital to home is an important healthcare system priority. This paper reports on the qualitative findings from a larger mixed methods study designed to examine the implementation and effectiveness of a new transitional care intervention (Community Assets Supporting Transitions [CAST]). The goal of the CAST intervention is to improve the quality and experience of hospital-to-home transitions for older adults (≥ 65 years) with depressive symptoms and multimorbidity. Semi-structured interviews were completed with a sub-set of intervention group trial participants including 11 older adult participants and 1 caregiver, as well as 4 intervention nurses. A qualitative descriptive design was used to explore the perceived impacts of the CAST intervention on participants and their caregivers. Audio-recorded interviews were transcribed verbatim, with descriptive codes and themes generated using conventional content analysis. Patient participants indicated that the intervention resulted in improved access to information (e.g., medication review) and services (e.g., care coordination) that enhanced their self-management. Participants felt that the home visits and phone visits were valuable and helped to improve their mental health. Intervention nurses described advocating for patients to help achieve their needs. For example, nurses advocated for physiotherapy services to provide additional education to support patient mobility. Understanding patient, caregiver, and provider perceptions of the impact of the CAST intervention will help to identify how to improve the delivery of this transitional care intervention, to bridge the gap between hospital and community care, and to positively impact patient health outcomes.

